# Discovery of Potential Inhibitors of Squalene Synthase from Traditional Chinese Medicine Based on Virtual Screening and In Vitro Evaluation of Lipid-Lowering Effect

**DOI:** 10.3390/molecules23051040

**Published:** 2018-04-28

**Authors:** Yankun Chen, Xi Chen, Ganggang Luo, Xu Zhang, Fang Lu, Liansheng Qiao, Wenjing He, Gongyu Li, Yanling Zhang

**Affiliations:** 1 School of Chinese Material Medica, Beijing University of Chinese Medicine, Beijing 100102, China; 18811791975@163.com (Y.C.); chenxi_cx95@163.com (X.C.); 17801080765@163.com (G.L.); 18003381008@163.com (X.Z.); lufang1017@163.com (F.L.); b20100222012@163.com (L.Q.); lidoc2727@163.com (G.L.); 2 College of Traditional Chinese Medicine Xinjiang Medical University, Urumqi 830054, China; wenjhe@163.com

**Keywords:** hyperlipidemia, squalene synthase (SQS), molecular modeling, drug discovery, Traditional Chinese Medicine

## Abstract

Squalene synthase (SQS), a key downstream enzyme involved in the cholesterol biosynthetic pathway, plays an important role in treating hyperlipidemia. Compared to statins, SQS inhibitors have shown a very significant lipid-lowering effect and do not cause myotoxicity. Thus, the paper aims to discover potential SQS inhibitors from Traditional Chinese Medicine (TCM) by the combination of molecular modeling methods and biological assays. In this study, cynarin was selected as a potential SQS inhibitor candidate compound based on its pharmacophoric properties, molecular docking studies and molecular dynamics (MD) simulations. Cynarin could form hydrophobic interactions with PHE54, LEU211, LEU183 and PRO292, which are regarded as important interactions for the SQS inhibitors. In addition, the lipid-lowering effect of cynarin was tested in sodium oleate-induced HepG2 cells by decreasing the lipidemic parameter triglyceride (TG) level by 22.50%. Finally. cynarin was reversely screened against other anti-hyperlipidemia targets which existed in HepG2 cells and cynarin was unable to map with the pharmacophore of these targets, which indicated that the lipid-lowering effects of cynarin might be due to the inhibition of SQS. This study discovered cynarin is a potential SQS inhibitor from TCM, which could be further clinically explored for the treatment of hyperlipidemia.

## 1. Introduction

Hyperlipidemia, characterized by abnormally-elevated levels of cholesterol in the blood, is one of the main risk factors for atherosclerosis and visceral obesity [1]. Reduction of cholesterol can be achieved by inhibiting cholesterol biosynthesis [2]. To date, human HMG-CoA reductase (hHMGR) inhibitors such as statins are the most effective medicines for reducing cholesterol levels. However, these statins have potential adverse effects, such as myotoxicity, hepatotoxicity and even rhabdomyolysis [3]. The major cause of these side effects is the inhibition of HMG-CoA reductase that will interfere with the synthesis of many nonsteroidal isoprenoid molecules, which plays a major role in diverse cellular functions [4]. Compared to HMG-CoA reductase, squalene synthase (SQS), a key downstream enzyme involved in the cholesterol biosynthetic pathway, is regarded as an attractive target for anti-hyperlipidemia [5]. SQS is the first step of the steroid synthesis pathway, which means the inhibition of SQS can prevent the cholesterol biosynthesis without interrupting isoprenoid production [6]. Due to its strategic location in the pathway, inhibitors of SQS are promising drugs for the treatment of hyperlipidemia. 

At present, chemical synthesis [7] and genetic engineering methods [8] are utilized to discover SQS inhibitors, which requires much time and money. Traditional Chinese Medicine (TCM) has been widely used in the treatment of hyperlipidemia with low cost and minimal adverse effects. For example, *Fructus Crataegi* and *Salviae Miltiorrhizae* are the most well-known used Chinese herbs for treating hyperlipidemia [9,10]. Although TCM has played an important role in drug discovery for treating hyperlipidemia for a long time due to its rich natural resources, there are few studies at present on the discovery of SQS inhibitors from TCM. Thus, it is of great importance to discover potential SQS inhibitors from TCM. In [11] the authors researched SQS inhibitors by using molecular docking and virtual screening methods but the shortcoming of the study was the lack of biological assays to verify the accuracy of the results. 

In our study, we provide a reliable strategy to discover potential SQS inhibitors from TCM by the combination of molecular modeling methods and biological assays. First, ten HipHop pharmacophore models were generated based on known SQS inhibitors. The optimal pharmacophore model was selected by four validation indices and used as a query to screen potential SQS inhibitors from the Traditional Chinese Medicine Database (TCMD, Version 2009). Molecular docking was employed to refine the pharmacophore model hits and analyze the protein-ligand binding modes. Then, MD simulations were performed to validate the binding stability between the compounds and the protein. The potential SQS inhibitors were selected based on the fitvalue, docking score, and interactions formed between the ligands and SQS. In addition, the compounds were evaluated for the lipid-lowering effect in sodium oleate-induced HepG2 cells. Finally, the active compounds were utilized to reversely identify the other anti-hyperlipidemia targets existed in HepG2 cells to further evaluate the lipid-lowering effect was due to the inhibition of SQS. This study aims to discover potential SQS inhibitors from TCM, which also provide the candidate compounds for the clinical treatment of hyperlipidemia. 

## 2. Results

### 2.1. Pharmacophore Model Studies

Ten pharmacophore models were generated based on twenty-two SQS inhibitors by the HipHop method within the Discovery Studio 4.0 (DS) from Accelrys (San Diego, CA, USA). All of the models had high rank scores (154.43–157.40, Table 1), which indicated that compounds in the training set mapped well with generated pharmacophore models. The test set was applied for evaluating the generated ten pharmacophore models based on the three evaluation indices as follows: hit rate of active compounds (*HRA*), identify effective index (*IEI*) and comprehensive appraisal index (*CAI*). *HRA*, *IEI* and *CAI* are defined by Equations (1)–(3), where D represents the total number of compounds in the test set and A represents the number of active compounds in the test set. Ht is the total number of hit compounds from the test set and Ha represents the number of active hit compounds from the test set. *HRA* represents the ability to identify active compounds from the test set. *IEI*, the index of effective identification, is used to evaluate the ability of the models to identify active compounds from the inactive compounds. *CAI* is the comprehensive evaluation of pharmacophore model [12]:

(1) $$HRA=\left(\frac{\mathrm{Ha}}{A}\right)\times 100$$

(2) $$IEI=\frac{\left(\frac{\mathrm{Ha}}{\mathrm{Ht}}\right)}{\frac{A}{D}}$$

(3) CAI=HRA×IEI

**Figure 1:**
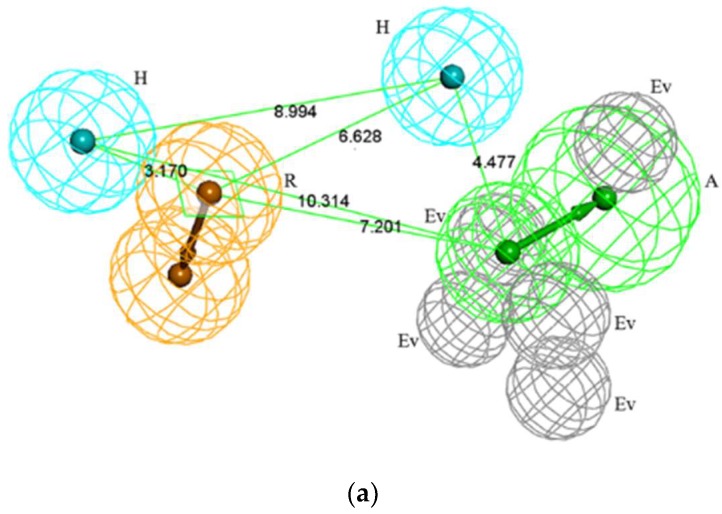

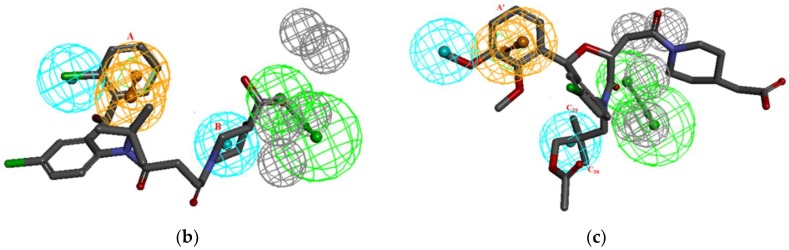
(**a**) The optimal pharmacophore model Hypo1; Wherein, green features represent hydrogen bond acceptor (A), light blue features represent hydrophobic features (H), orange features represent ring aromatic (R) and gray features represent excluded volumes (Ev); (**b**) The mapping of the crystallographic ligand with the optimal pharmacophore model Hypo1; (**c**) mapping of TAK-475 with the Hypo1.

The evaluation results of the 10 pharmacophore models are shown in Table 1. The calculation of the *HRA* index returned values greater than 80% for nine of 10 models, revealing the high accuracy of the generated pharmacophore models. The rank score represents the total score of how the training set fits the pharmacophore, and the best model has the highest rank [13]. Hypo1 had the highest rank score of 157.40. Therefore, Hypo1 was selected as the optimal pharmacophore model. In general, scores of *HRA*, *IEI* and *CAI* above the values of 80%, 2, and 2 are considered excellent. *HRA*, *IEI* and *CAI* of Hypo1 were 94.16%, 2.26, and 2.12, respectively. As shown in Figure 1a, Hypo1 contained one hydrogen bond acceptor (A), two hydrophobic features (H), one aromatic ring (R), and five excluded volumes (Ev). In order to validate the veracity of the best pharmacophore model, the crystallographic ligand of D99 and the positive SQS inhibitor of TAK-475 [14] were mapped with the optimal pharmacophore model. Both compounds mapped well with all the features of Hypo 1, which are shown in Figure 1b,c. 

According to the literature, researchers have constructed pharmacophore models of SQS [15,16]. We further compared our pharmacophore model to those of these researchers. First, the method used for constructing the pharmacophore model was different. The pharmacophore models in the literature were constructed by using the three-dimensional quantitative structure-activity relationship (3D-QSAR) method, which belongs to the quantitative hypothesis models, while we built the pharmacophore models by using HipHop method, which belongs to the qualitative hypothesis models. Second, the structure of the training sets was different. The structures of the training set in the articles were relatively simple, aimed at directing the structural modification of the potential compounds. Our training set with structural diversity was used to screen active compounds with novel structures from the database. Third, the purposes of the papers were different. The researchers used a training set of ligands with activity values to derive 3D-QSAR pharmacophore models for prediction. Our HipHop pharmacophore was built by using a training set of some active ligands to derive common feature pharmacophores for lead identification. Fourth, the similarity analysis. The features of the 3D-QSAR pharmacophore model and the HipHop pharmacophore such as hydrogen bond acceptor, hydrophobic features, aromatic ring, and excluded volumes, were consistent, which indicated that our HipHop pharmacophore was reliable and could be applicable to screen potential SQS inhibitors.

What is more, to further evaluate the reliability of the pharmacophore model, a 2D similarity search was used to compare the similarity between the TAK-475 and the 22 ligands used in the construction of the pharmacophore model based on 2D fingerprints [17]. During this process, the positive SQS inhibitor of TAK-475 as the template molecule was chosen to search for similar molecules in the 22 ligands, as the top-ranked molecules are likely to exhibit similar biological activity [18]. The Tanimoto coefficient [19] was used to measure the similarity to find ligands that are similar to TAK-475. In general, the range of Tanimoto coefficient values is from zero to one. A value closer to one indicates a greater similarity between the ligand and TAK-475. There is no specific standard for the threshold of Tanimoto coefficient to identify ligands, the Tanimoto coefficient value of 0.3 was also set as threshold in some references to identify ligands [20]. From the results (Table 2), the 22 ligands had Tanimoto coefficient values all higher than 0.45. In addition, the ligands with Tanimoto coefficient values higher than 0.7 account for more than 60% of the 22 ligands. The result indicated that these 22 ligands had similar structures compared to TAK-475, with similar biological activity and could be used to construct the pharmacophore model. 

Then the Hypo 1 program was used to screen potential SQS inhibitors from the Traditional Chinese Medicine Database (TCMD, Version 2009), before which the TCMD database was filtered based upon the Lipinski’s rules, leaving 13,905 compounds. Then, a hit list of 1775 TCM compounds were obtained for further docking studies.

### 2.2. Molecular Docking Studies

**Figure 2:**
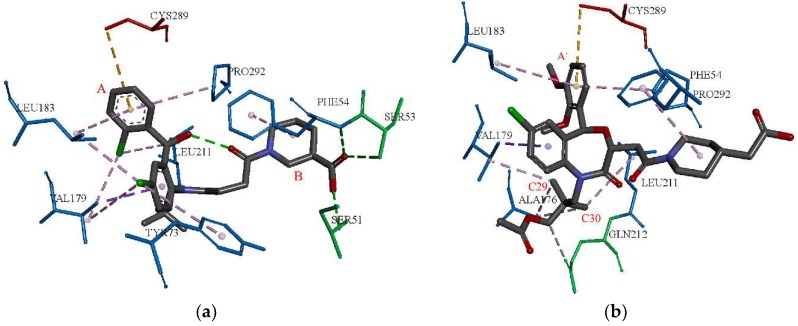
(**a**) the docking result of the crystallographic ligand with the crystal structure of SQS; (**b**) the docking result of TAK-475; the pink dash line represented hydrophobic effect; the green dash line represented hydrogen bond donor; the green amino acids represent hydrogen bond interactions; blue amino acids represent hydrophobic interactions.

The binding pocket was defined with a default parameter of sphere radius of 9.16 Å around D99 of SQS. The D99 was re-docked into the active pocket by using two docking algorithms, LibDock and CDOCKER, respectively. The RMSD values of D99 were 7.98 Å and 0.69 Å for the corresponding two docking algorithms. The reason for such a high RMSD returned by LibDock, in comparison to CDOCKER, may be ascribed to the differences between the two docking algorithms. LibDock is a kind of semi-flexible docking method and CDOCKER is a flexible one. In addition, the LibDOCK algorithm is a high-throughput algorithm for docking ligands into receptor binding sites [21]. The CDOCKER algorithm uses a CHARMm-based molecular dynamics (MD) method to dock ligands into an active receptor site [22]. The ligand can generate random conformations to form a favorable interaction with the protein, which may cause a lower RMSD compared to LibDock. In general, an RMSD less than 2.00 Å shows that the docking algorithm is fit for this protein-ligand binding mode. The closer the RMSD is to zero, the better is the docking result [23]. Therefore, the CDOCKER algorithm is appropriate and employed to perform molecular docking studies. The CDOCKER energy (kcal/mol) and CDOCKER interaction energy (kcal/mol) of D99 were 51.30 and 61.78, respectively, which were the scoring function of the CDOCKER algorithm. The CDOCKER energy indicated the energy of the ligand-protein complexes, and the CDOCKER interaction energy represented the energy of the ligands [24]. The interaction between the D99 and the protein was analyzed in detail, which is shown in Figure 2a. D99 could form hydrogen bond interactions with PHE54, SER51, ARG52, SER53, and generated hydrophobic interactions with PHE54, TYR73, VAL179, LEU183, LEU211, and PRO292. 

TAK-475 was then successfully docked into the active pocket, which further indicated the docking model was reasonable. The CDOCKER energy and CDOCKER interaction energy of TAK-475 were 55.34 and 74.39, which were both higher than the scores of D99. The interaction between TAK-475 and the active site was further analyzed. TAK-475 formed the hydrogen bond interactions with GLN212, and formed the hydrophobic interactions with PHE54, ALA176, VAL179, LEU183, LEU211, and PRO292 (shown in Figure 2b). D99 and TAK-475 both formed hydrophobic interactions with PHE54, VAL179, LEU183, LEU211, and PRO292. Thus, these amino acids were regarded as key residues, which is consistent with the literature [25,26]. 

**Figure 3:**
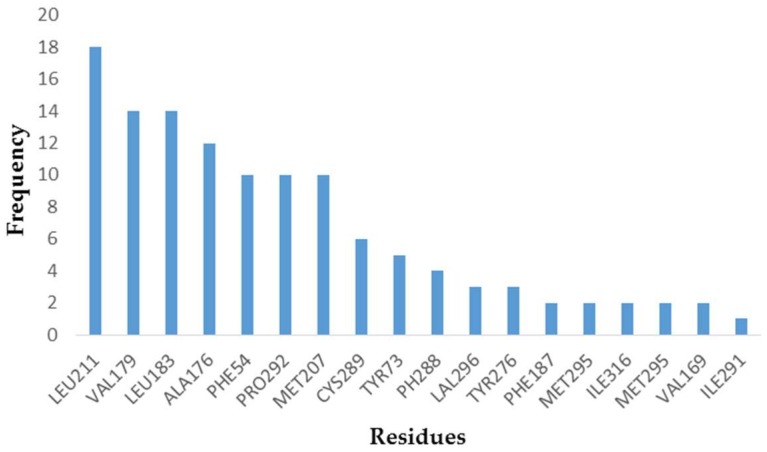
The frequency of hydrophobic amino acids formed by 22 compounds.

After that, the 22 ligands used in the construction of the pharmacophore model were docked into the binding site of SQS for further demonstrating the key amino acids in receptor-ligand interaction. By counting the frequency of hydrophobic amino acids formed by 22 compounds, the receptor-ligand hydrophobic interactions column diagram shown in Figure 3 was generated. From the result, most of the active compounds could form the hydrophobic interactions with LEU211, VAL179, LEU183, ALA176, PHE54, PRO292, and MET207. This indicated that D99, TAK-475 and the 22 active compounds all could form hydrophobic interactions with PHE54, VAL179, LEU183, LEU211, and PRO292, which were considered to be important key amino acids and used as the reference for selecting potential inhibitors.

Then 1775 drug-like characteristic compounds which were filtered by the optimal pharmacophore model and Lipinski’s rules were docked into the binding pocket of SQS. The threshold of the docking score, which is mentioned in the material section of molecular docking, was used to select the potential compounds, and then a hit list of 37 compounds was obtained. Among the 37 potential compounds, cynarin, which got the high docking score and formed an important binding mode with SQS was considered as the most promising candidate. 

**Figure 4:**
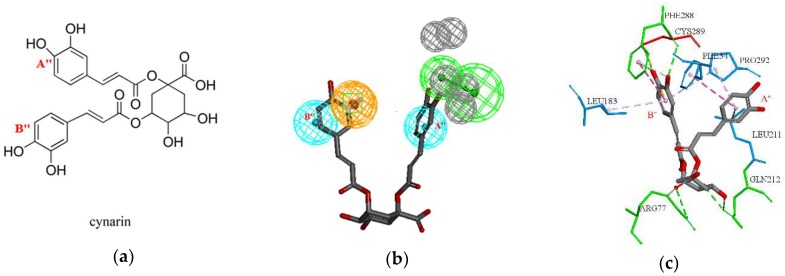
(**a**) The 2D structures of cynarin; (**b**) The mapping results of cynarin with Hypo1; (**c**) the docking result of cynarin with the crystal structure of SQS; the green amino acids represent hydrogen bond interactions; blue amino acids represent hydrophobic interactions.

More specifically, cynarin obtained a CDOCKER energy of 42.08 and CDOCKER interaction energy of 52.92, and formed hydrogen bond interactions with PHE288, GLN212, ARG77, and CYS289, and hydrophobic interactions with PHE54, LEU183, LEU211, and PRO292. The details are shown in Figure 4. In addition, the docked pose of cynarin was screened with the pharmacophore model to further ensure the docked pose fit the pharmacophore model. The result indicated that cynarin was mapped with three features of the optimal pharmacophore model and the fitvalue was 0.66. Moreover, one benzene ring A’’ of cynarin could form hydrophobic interactions with PHE54, LEU211, and PRO292, which mapped with one H feature in the pharmacophore model. Another benzene ring B’’ of cynarin formed hydrophobic interactions LEU183 and PRO292, and also mapped with another H feature in the pharmacophore. Compared with D99 and TAK-475, cynarin formed similar hydrophobic interactions with PHE54, LEU183, LEU211, and PRO292. Moreover, the features contained in the pharmacophore model of Hypo1 and the specific hydrophobic interactions formed between cynarin and SQS were consistent. The rationality of our pharmacophore model and molecular model were also confirmed. 

### 2.3. MD Simulations

**Figure 5:**
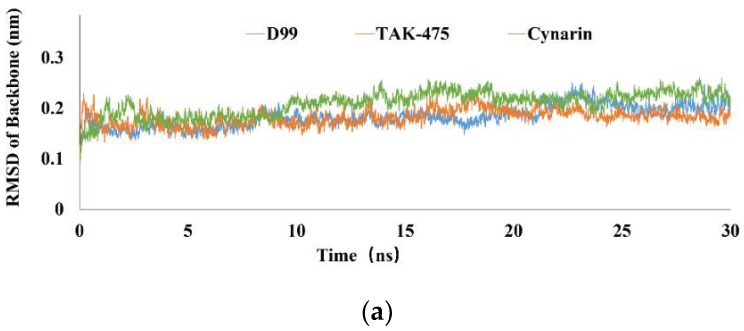

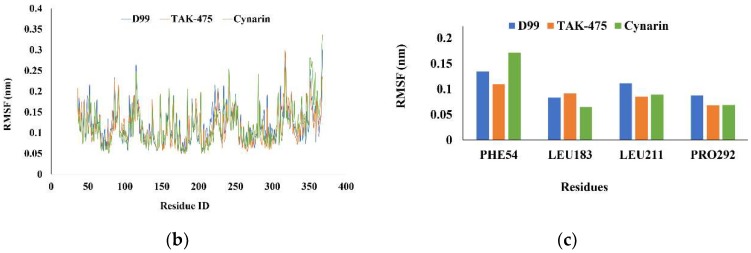
(**a**) The trajectory of MD simulations of three complexs: average protein RMSD; Blue, red and green bars represent for the data of D99, TAK-475 and cynarin, respectively; (**b**) Root mean square fluctuation (RMSF) corresponds to MD trajectory; (**c**) the analysis of hydrophobic residues implicated in docking.

MD simulations were implemented to analyze the binding stability of SQS-cynarin, SQS-D99, and SQS-TAK-475 under dynamic conditions. The RMSD of the protein backbone of each protein-ligand complex were calculated to evaluate the stability of the system [27]. The RMSD trajectories of the SQS-cynarin, SQS-D99 and SQS-TAK-475 complexes were equilibrated after 15 ns (shown in Figure 5a). The root mean square fluctuation (RMSF) was further calculated to evaluate the flexibility of the residues. The results were plotted using residue numbers at the simulation trajectory, which is shown in Figure 5b. It can be observed that the SQS-cynarin complex exhibited a similar RMSF value in comparison to the SQS-D99 and SQS-TAK475 complexes. The protein residues with lower RMSF value are regarded as more stable [28]. Then, by analyzing the flexibility of the important hydrophobic residues, including PHE54, LEU183, LEU211, and PRO292, these amino acids in the cynarin complex had similar RMSF values as in the D99 and TAK-475 complexes (shown in Figure 5c), which were regarded as important and stable hydrophobic interactions between cynarin and SQS. 

Then the binding free energy of the SQS-cynarin, SQS-D99, and SQS-TAK-475 complexes was calculated by the Molecular Mechanic-Poisson Boltzmann Surface Area (MM-PBSA) with GROMACS v5.0.2 [29], with the results listed in Table 3. The results indicated that SQS-cynarin, SQS-D99, and SQS-TAK-475 complexes possessed a negative binding free energy of −210.39, −253.03 and −285.36 kJ/mol. Moreover, van der Waals, electrostatic interactions and non-polar solvation energy negatively contributed to the total interaction energy, while only polar solvation energy positively contributed to total free binding energy. Thus, the relative binding free energies of the SQS-cynarin, SQS-D99, and SQS-TAK-475 complexes indicated the strong binding in the dynamic system. To obtain a more detailed thermodynamic description of the residue contributions to the binding free energy, we decomposed the binding energy ΔG_MM-GBSA_ on a per-residue level depicted in Table 4. The contribution of residue PHE54, LEU183, LEU211, and PRO292 to binding varies from −2.32 to −11.56 kJ/mol, which could be identified as the key residues of SQS. Based on the consensus results among the pharmacophore based virtual screening and the docking/MD simulations, cynarin exhibited a key and stable interaction profile with SQS, being regarded as a potential SQS inhibitor. 

### 2.4. Experimental Result

**Figure 6:**
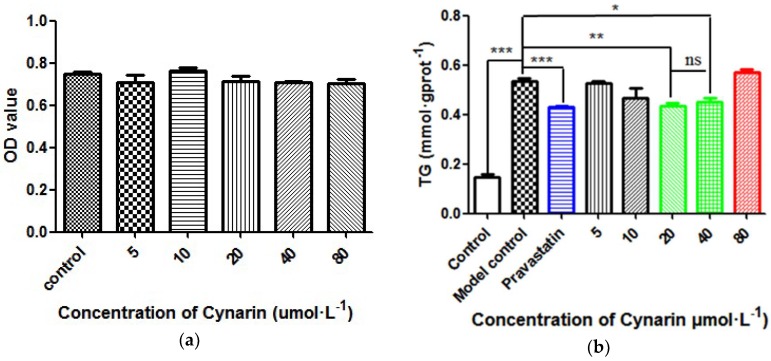
(**a**) Cell-viability of different concentration of cynarin on HepG2 cells by the MTT assay; (**b**) Effect of different concentration of cynarin on the TG content in sodium oleate-induced HepG2 cells (* means *p* <0.05, ** means *p* < 0.01 and *** means *p* < 0.001 compared with the model control group).

To test the lipid-lowering effect of cynarin (CAS number: 19870-46-3), sodium oleate-induced HepG2 cells were treated with various doses of cynarin (5, 10, 20, 40, and 80 μmol·L^−1^), and the positive compound pravastatin, respectively. The control group cells were cultured with only HepG2 cells. The model control group cells were the hyperlipidemia cell model. The positive control group cells were cultured with pravastatin. Firstly, the MTT assay was utilized for the detection of cell viability, with the result shown in Figure 6a. From the result, the five different concentrations of cynarin were not cytotoxic to HepG2 cells compared to the control group (*p* > 0.05).

Then, the lipid-lowering effect of cynarin was evaluated in sodium oleate-induced HepG2 cells, which is shown in Figure 6b. Compared to the control group, the plasma triglyceride (TG) level of the model control group shows a significant difference with the control group (*p* < 0.001), which indicates that the hyperlipidemia cell model could be used for evaluating the lipid-lowering activity of cynarin. In addition, the pravastatin could decrease the TG level compared to the model group (*p* < 0.001), which demonstrated the hyperlipidemia cell model was reliable. From the result, 20 μmol·L^−1^ cynarin and 40 μmol·L^−1^ cynarin could both decrease the TG level, and there was no difference between these two groups in statistics (*p* > 0.05). However, the result of 20 μmol·L^−1^ cynarin for reducing the TG level was more reliable with a higher confidence interval (*p* < 0.01) compared to 40 μmol·L^−1^ cynarin (*p* < 0.05). Thus, the optimum concentration of cynarin was 20 μmol·L^−1^, which could decrease the TG level by 22.50%. Cynarin was mildly cytotoxic to the sodium oleate-induced HepG2 cells at 80 μmol·L^−1^, so it may be speculated that the sodium oleate-induced HepG2 cells were more sensitive compared to normal HepG2 cells. On the basis of the above analysis, cynarin could be a potential SQS inhibitor for the treatment of hyperlipidemia. 

Cynarin, also called 1,3-dicaffeoylquinic acid, was identified as a potential SQS inhibitor. Cynarin is a common component of various TCM herbs such as *Cynara scolymus*, *Cynara cardunculus*, and *Senecio nemorensis*. It was proved to have positive pharmacological choleretic, hepatoprotective, anti-atherosclerotic, anti-oxidant, anti-cholinergic, antioxidative, anticarcinogenic effects and so on. To be specific, for the anti-atherosclerotic effects, the researchers demonstrated that cynarin could reduce the nitric oxide synthase (iNOS) activity and cynarin was the most effective with 3 µM [30]. For the hepatoprotective effects, the study with the rat hepatocytes indicated that 3 µM cynarin could reduce *tert*-butylhydroperoxide (t-BPH)-induced malondialdehyde (MDA) production and EC50 value of cynarin was 15.2 µg/mL [31]. For the anti-diabetic effects, the study demonstrated the potential antiglycative effects of cynarin in the bovine serum albumin-glycose system, and cynarin could inhibit the ability of advanced glycation end products (AGE) in a dose dependent manner (3 µM–40 µM) [32]. Meanwhile, consulting the literature, there are no reports about drug interactionz between cynarin and other SQS inhibitors. Combining these results with our research, cynarin was proved to be a potential SQS inhibitor, and in view of the extremely low toxicity of the cynarin, which provided a new perspective for the treatment of hyperlipidemia.

### 2.5. Anti-Hyperlipidemia Target Identification by Pharmacophore

To provide more evidence for the lipid-lowering effect of cynarin on SQS activity at the molecular level, cynarin was utilized to reversely screen it against the pharmacophore models of other anti-hyperlipidemia targets that exist in HepG2 cells. The fitvalue was used as an important judgment index to represent the overlap degree between the compound and pharmacophore model [33]. According to the screening results, cynarin was unable to map with the pharmacophore models of these commonly used targets, including 3-hydroxy-3-methylglutaryl coenzyme A (HMG-CoA) [34], peroxisome proliferator-activated receptor-α (PPAR-α) [35], liver X receptor β (LXRβ) [36], cholesteryl ester transfer protein (CETP) [37], and microsomal triglyceride transfer protein (MTP) [37], which is shown in Figure S1. The result indicated that the lipid-lowering effects in HepG2 cells of cynarin might due to the inhibition of SQS. In addition, based on the above results, cynarin is regarded as a promising SQS inhibitor candidate and could be explored for the treatment of hyperlipidemia. The biological activity of cynarin against other targets should also be studied in the future research.

## 3. Materials and Methods 

### 3.1. HipHop Pharmacophore Hypotheses Generation

**Figure 7:**
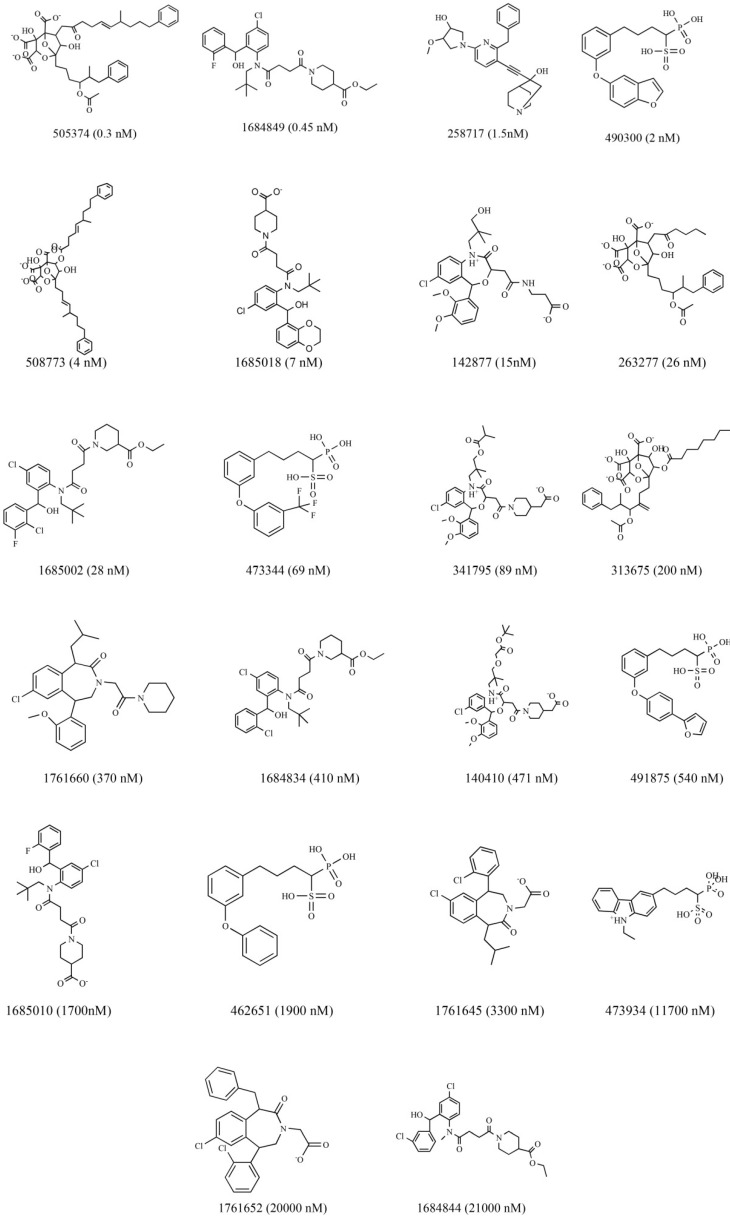
Structures, ID and the value of IC_50_ of 22 compounds in the training set for pharmacophore model generation of SQS.

Among the library compounds 22 active compounds were selected as the training set and were used to generate HipHop pharmacophore models by using DS 4.0 from Accelrys (San Diego, CA, USA). The structure, ID numbers, and biological activity (IC_50_) values of the compounds are shown in Figure 7. Then, 154 active compounds and 462 inactive compounds [38], which selected randomly from the Binding Database, were regarded as the test set in order to validate the pharmacophore model. The 3D structures of all the compounds were generated using the ‘Prepare Ligands’ module and minimized in CHARMm force field [39]. The conformations of these compounds were created within an energy threshold of 20 kcal/mol by using the BEST method. The maximum ligand conformations were set to 255.

The HipHop pharmacophore models were constructed by extracting the common pharmacological features from the 3D structure features of each compound in the training set [40]. The Principal and MaxOmitFeat values are used to describe the activity of the compounds. The range of “Principle” and “MaxOmitFeat” values are 0, 1 and 2. The “Principal” value is set to 2, representing the superior activity of the compounds. The corresponding “MaxOmitFeat value is set to 0, which indicates that no features that are allowed to be missed for each compound. Then, the “Principal” value is set to 0, indicating the lower activity of the compounds. 

The corresponding “MaxOmitFeat value” is set to 2 to suggest that all features can be ignored for these compounds [38]. The maximum excluded volumes (Ev) value was set to 5, and all the other parameters were set at default values. The optimal pharmacophore model was selected based on rank score, *HRA*, *IEI*, and *CAI.* Then, the crystallographic ligand and the positive SQS inhibitor TAK-475 were used to map the optimal model to further evaluate the accuracy of the pharmacophore model. In addition, in order to validate the reliability of the best pharmacophore model, based on 2D fingerprints, similarity search method was utilized to compare the similarity between the 22 ligands and the TAK-475. 

The selected optimal pharmacophore model was then utilized to screen potential SQS inhibitors from TCMD [41], before which the TCMD database was filtered based upon the Lipinski’s rules for drug–likeness prediction [42]. The list of compounds with drug-like characteristics was regarded as potential SQS inhibitors and were retained for molecular docking study. 

### 3.2. Molecular Docking Studies

The crystal structure of the human SQS (PDB entry 3ASX, resolution 2.0 Å) was obtained from the RCSB Protein Data Bank (PDB), which is complexed with an inhibitor, (3*R*)-1-{4-[{4-chloro-2-[(*S*)-(2-chlorophenyl)(hydroxy)methyl]phenyl}(2,2-dimethylpropyl)amino]-4-oxobutanoyl}piperidine- 3-carboxylic acid (D99) [43]. The protein was automatically cleaned up by the Prepare Protein protocol for some common problems, such as incomplete residues, the lack of hydrogens, the existence of crystallographic water and ligands [44]. The binding active pocket of 3ASX was determined around the crystallographic ligand using the Define and Edit Binding Site tools. LibDock and CDOCKER, two common docking algorithms, were utilized to evaluate the applicability for the docking studies. The crystallographic ligand D99 was extracted from the active pocket and was then re-docked into the crystal structure by these two docking methods. The docking algorithm with the smallest RMSD was used for the study. In addition, the positive SQS inhibitor TAK-475 was docked into the active pocket of SQS, which further evaluated the rationality of the docking model. Then the interactions between D99, TAK-475 and the active pocket of SQS were analyzed. 

After that, the 22 ligands used in the construction of the pharmacophore model were docked into the active binding pocket of SQS to further analyze the key amino acids. Then, the hit compounds screened by the optimal pharmacophore model were docked into the binding site. Eighty percent of the docking scores of D99 were regarded as the threshold value for identifying potential SQS inhibitors from TCMD [45]. Finally, the compounds which got a high docking scores and formed similar interactions to D99 and TAK-475 were obtained to evaluate the stability of the complex. 

### 3.3. MD Simulations

A 30 ns MD simulation was employed to investigate the dynamic binding stability of the complexes with GROMACS v5.0.2 using GROMOS96 43a1 force field [46]. Initially, the topology parameters of SQS were obtained using the GROMACS program and the force field parameters for the three ligands were derived from PRODRG server [47]. In each simulation, the complex was solvated using simple point charge (SPC) water molecules [48] and five sodium ions were added by replacing solvent molecules in order to neutralize the system. Each system consisted of ~22,800 waters molecules and the solvent and ions around the protein were first equilibrated before collecting frames for analysis. The energy minimizations were carried out using the steepest descent method with 5000 steps. The system was then subjected to two phases of equilibration for a period of 1500 ps at 300 K with position restraints on the protein and ligands (fc = 1000). A first 500 ps NVT equilibration was performed using V-rescale thermostat coupling method [49] for temperature control in order to relieve any bad contacts at the residues solvent interface [50]. Then a 1000 ps NPT equilibration was conducted at 1.0 bar using Parrinello-Rahman barostat method [50] for pressure control. Upon the two equilibration phases, the position restraints were released and MD simulations were produced. 

By consulting the related literatures, for example, the researchers performed a relatively short time (such as 10–30 ns) MD simulation to evaluate the binding stability during a dynamic environment and analyze the key amino acids by a series of MD analysis tools such as RMSF, RMSD and the total energy [51,52]. It makes sense and contributes to the whole paper for the discovery of the potential compounds. Actually, 30 ns might still be a little short, but literatures have showed it could also give key information for molecular modeling [53,54].

In addition, the MM-PBSA method has been widely utilized to study the receptor-ligand interaction. For the three complexes including SQS-cynarin, SQS-D99, and SQS-TAK-475 system, free energy calculations were performed for 10 snapshots extracted from the last 1 ns stable MD trajectory using g_mmpbsa tool [55]. The MM-PBSA method can be summarized by the following equations.

For each snapshot, the free energy was calculated for each molecular species (complex, protein and ligand) and the binding free energy was computed by Equation (4). The free energy of each component Gx in Equation (4) could be calculated taking in account three terms (Equations (5)–(8)):

ΔG_*binding*_ = G_*complex*_ − (G_*protein*_ + G_*ligand*_) (4)

G*_x_* = E*_MM_* + G*_solv_* − TΔS (5)

E*_MM_* = E*_vdW_* + E*_ele_* (6)

G_*solv*_ = G_*polar*_ + G_*nonpolar*_ (7)

G*_nonpolar_* = γSASA + β (8)

G*_MM_*, the molecular mechanics energy, was calculated by the electrostatic and van der waals interactions. G*_solv_*, the solvation free energy, was composed of the polar and the nonpolar contributions. Polar solvation free energy could be obtained by solving the Poisson-Boltzmann equation for MM/PBSA method, whereas nonpolar solvation free energy was determined using Solvent Accessible Surface Area (SASA) model. TΔS represents the entropy term.

### 3.4. Experimental Validation

The lipid-lowering activity of the potential compound was evaluated by examining the inhibition of the formation of lipid droplets in HepG2 cells in vitro. Bligh et al. [56] have reported an efficient and rapid method of total lipid extraction and purification. Compared with this method, we used sodium oleate-induced HepG2 cells to generate the lipid droplets [57]. The cells were grown at 37 °C with 5% CO_2_ in DMEM solution containing 10% FBS and 1% penicillin/streptomycin. 

Then cell viabilities were determined by 3-(4,5-dimethylthiazol-2-yl)-2,5-diphenyltetrazolium bromide (MTT) method [58]. In 96-well plates, HepG2 cells were seeded for 24 h at a density 2 × 10^4^ cells/well, and then incubated at various concentrations of the compounds for another 24 h. Then, each well was treated with 200 uL MTT working solution (5 mg·mL^−1^) and cultured for a further 4 h. After removing the MTT, 150 uL dimethylsulphoxide (DMSO) was added to each well for terminating response, and the plate was set to the table shaker for 5 min at a low speed. Then the absorbance of cells was measured at 570 nm using microplate reader. The maximum concentration of the compound that can be used for the assay was determined by the MTT cytotoxicity assay in HepG2 cells. 

To evaluate the lipid-lowering effect of the potential compounds, HepG2 cells were induced by sodium oleate for establishing a model of hyperlipidemia [59]. The HepG2 cells were seeded in 6-well plates at 20 × 10^4^ cells/well for 24 h. Then, sodium oleate was added into the each well for producing fat accumulation as model cells at 60 µg/mL concentration and incubated at another 24 h. The control group cells were cultured without sodium oleate. It has been proved that SQS inhibitors could reduce TG level through an LDL receptor-independent mechanism [60]. Tavridou et al. [61] demonstrated that SQS inhibitors could significantly reduce the TG level in HepG2 cells. Moreover, other related literature has indicated that SQS inhibitors can decrease the TG level in in vivo experiments [26,62]. To measure the lipidemic parameter triglyceride (TG) level, appropriate kits were utilized to analyze the TG content in HepG2 cells.

### 3.5. Anti-Hyperlipidemia Target Profiling

Ligand profiler module is an important method to reversely identify the action targets for candidate compound, and it is widely used for drug poly-pharmacology prediction of TCM [63]. In order to further illustrate the lipid-lowering effects of the active compound was caused by the inhibition of SQS at the molecular level, a pharmacophore database of other anti-hyperlipidemia targets, which exist in HepG2 cells, was built to assess the activity of the candidate. This anti-hyperlipidemia database contained five commonly used targets, including HMG-CoA, PPAR-α, LXRβ, CETP and MTP. Initially, diverse conformations of the active compound were generated by BEST mode with 255 conformations, and the relative energy threshold was less than 20.0 kcal/mol. The generated conformations were regarded as query to map with the anti-hyperlipidemia pharmacophore database by flexible searching method. 

## 4. Conclusions

The main purpose of this study was to screen potential SQS inhibitors from Chinese herbs using a series of methods, including molecular modeling methods including pharmacophore model, molecular docking, MD simulations, lipid-lowering experiments in HepG2 cells, and anti-hyperlipidemia target profiling. From the result, cynarin, with high fitvalue, docking scores and predicted to form similar and stable interactions with SQS (as suggested by the MD simulations) was selected as a potential SQS inhibitor. Then, cynarin was investigated for its lipid-lowering effect on sodium oleate-induced HepG2 cells, and it was shown to decrease the lipidemic parameter triglyceride (TG) level by 22.50% using appropriate kits. Finally, to provide more evidence for the lipid-lowering effect of cynarin on SQS activity, cynarin was utilized to reversely identify other anti-hyperlipidemia targets existing in HepG2 cells, where it was unable to map with pharmacophores of these targets, which indicated that lipid-lowering effect of cynarin was due to the inhibition of SQS to some extent. 

By the combination of three different computational approaches and biological assays, cynarin was selected as a potential SQS inhibitor and could be explored for the treatment of hyperlipidemia. Furthermore, the established assay of sodium oleate-induced steatosis on HepG2 cells provided a rapid method for evaluating the lipid-lowering effect of other compounds. According to the related literature, it is very difficult and complex to obtain and purify the SQS protein. With the development of biological experimental technique, the follow-up study can be a further validation that cynarin actually targets SQS by western blotting. In conclusion, this study provided a promising SQS inhibitor candidate compound for the treatment of hyperlipidemia. The combination of computational approaches and biological assays contributed to the discovery of active compounds from TCM.

## Figures and Tables

**Table 1:** The Validation Results of the Pharmacophore Models.

Hypo	Feature	Rank	D	A	Ha	Ht	*HRA*	*IEI*	*CAI*
1	RHHAEv5	157.40	616	154	145	256	94.16%	2.26	2.12
2	RHHAEv5	156.97	616	154	147	290	95.45%	2.03	1.93
3	RHHAEv5	156.45	616	154	138	271	89.61%	2.04	1.83
4	RHHAEv5	155.73	616	154	138	278	89.61%	1.99	1.78
5	RHHAEv5	155.62	616	154	147	265	95.45%	2.22	2.12
6	RHHAEv5	155.54	616	154	151	268	98.05%	2.25	2.21
7	RHHAEv5	154.89	616	154	106	247	68.83%	1.72	1.18
8	RHHAEv5	154.67	616	154	126	219	81.81%	2.30	1.88
9	RHHAEv5	154.43	616	154	144	267	93.50%	2.16	2.02
10	RHHAEv5	154.43	616	154	143	254	92.86%	2.25	2.09

Note: D is the total number of compounds in test set; A is the number of active compounds in the test set; Ha is the hits number of active molecules mapped pharmacophores; Ht is the total hits number of molecules mapped pharmacophores; *HRA* (hit rate of active compounds); *IEI* (identify effective index); *CAI* (comprehensive appraisal index).

**Table 2:** Similarity search results of 22 ligands.

Tanimoto Coefficient ^a^	Number ^b^	Percent ^c^
0 < T ≤ 0.4	0	0
0.4 < T ≤ 0.5	4	0.18%
0.5 < T ≤ 0.7	4	0.18%
0.7 < T ≤ 0.8	7	0.32%
0.8 < T < 0.9	5	0.23%
0.8 < T < 1.0	2	0.09%

^a^ The Tanimoto coefficient is a similarity index. ^b^ Number is the number of ligands of the training set within in the corresponding threshold value of the Tanimoto coefficient. ^c^ Percent is percentage of the number of ligands.

**Table 3:** The binding free energy (kJ/mol) of the three complexes.

Complex	Binding Energy	Van der Waal Energy	Electrostattic Energy	Polar Solvation Energy	SASA Energy
SQS-cynarin	−210.39 ± 11.00	−291.56 ± 10.01	−39.10 ± 1.36	144.01 ± 0.25	−23.83 ± 0.61
SQS-D99	−253.03 ± 4.59	−310.59 ± 13.49	−36.47 ± 1.89	118.63 ± 6.81	−24.60 ± 0.20
SQS-TAK-475	−285.36 ± 6.50	−374.76 ± 7.76	−32.18 ± 0.97	149.79 ± 1.23	−28.20 ± 0.95

**Table 4:** The contribution of residues to binding free energy (kJ/mol).

Complex	PHE54	LEU183	LEU211	PRO292
SQS-cynarin	−10.56 ± 0.90	−2.32 ± 1.04	−11.55 ± 0.32	−5.40 ± 0.26
SQS-D99	−11.56 ± 0.53	−5.04 ± 0.53	−10.15 ± 0.95	−9.44 ± 1.13
SQS-TAK-475	−7.68 ± 0.49	−6.15 ± 0.10	−11.42 ± 1.39	−8.69 ± 0.96

## Supplementary Materials

The following are available online at http://www.mdpi.com/1420-3049/23/5/1040/s1, Figure S1.
