# Supplementary material for: Discovery of Potential Inhibitors of Squalene Synthase from Traditional Chinese Medicine Based on Virtual Screening and In Vitro Evaluation of Lipid-Lowering Effect
Source: Molecules. 2018 Apr 28;23(5):1040. doi: 10.3390/molecules23051040 (PMC6102583; doi:10.3390/molecules23051040)
Supplement: Supplementary file 1 [file molecules-23-01040-s001.pdf]

molecules

MDPI

Article

Synthase from Traditional Chinese Medicine Based on Virtual Screening and In Vitro Evaluation of Lipid-Lowering Effect

Yankun Chen [1], Xi Chen [1], Ganggang Luo [1], Xu Zhang [1], Fang Lu [1], Liansheng Qiao [1], Wenjing He [2], Gongyu Li [1] and Yanling Zhang [1,*]

[1] School of Chinese Material Medica, Beijing University of Chinese Medicine, Beijing 100102, China; 18811791975@163.com (Y.C.); chenxi_cx95@163.com (X.C.); 17801080765@163.com (G.L.); 18003381008@163.com (X.Z.); lufang1017@163.com (F.L.); b20100222012@163.com (L.Q.); lidoc2727@163.com (G.L.)

[2] College of Traditional Chinese Medicine Xinjiang Medical University, Urumqi 830054, China; wenjhe@163.com

* Correspondence: zhangyanling@bucm.edu.cn; Tel.: +86-10-8473-8620

Received: 19 March 2018; Accepted: 25 April 2018; Published: 28 April 2018

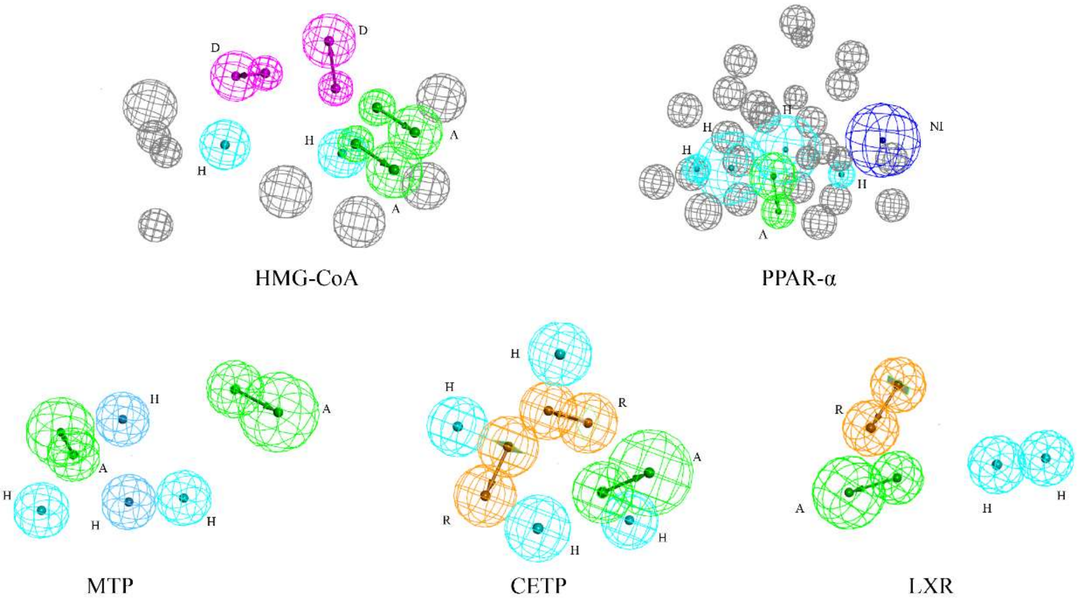

Figure S1. Anti-hyperlipidemia database, including 3-hydroxy-3-methylglutaryl coenzyme A (HMG-CoA), Peroxisome proliferator-activated receptor-α (PPAR-α), Liver X recptor β (LXRβ), Cholesteryl ester transfer protein (CETP), Microsomal triglyceride transfer protein (MTP), Wherein, green features represented hydrogen bond acceptor(A); pink features stood for hydrogen bond donor(D); dark blue features indicated negative ionizable group(NI); light blue features represented hydrophobic group(H); and orange features represented ring aromatic group(R).

© 2018 by the authors. Licensee MDPI, Basel, Switzerland. This article is an open access article distributed under the terms and conditions of the Creative Commons Attribution (CC BY) license (http://creativecommons.org/licenses/by/4.0/).
